# Examining *E.coli* aspartate transcarbamoylase with high-pressure crystallography

**DOI:** 10.1063/4.0001072

**Published:** 2025-10-27

**Authors:** Neti Bhatt M.S., Jaidyn Duhon, Michael Patterson, Stephen Meisburger, Nozomi Ando

**Affiliations:** 1 Cornell University, Ithaca, NY, USA; 2 Cornell High Energy Synchrotron Source, Ithaca, NY, USA

## Abstract

Many key biochemical pathways are regulated allosterically. High-resolution structure determination using macromolecular X-ray crystallography (MX) is a powerful method to elucidate allosteric mechanisms. However, a key limitation of MX is that structural snapshots may not capture the relevant conformational dynamics. This limitation may be overcome by applying perturbations, such as temperature and pressure, thus giving a more complete picture of the conformational landscape. In this study, we use high-pressure MX (HP-MX) to study the allosteric enzyme *E.coli* aspartate transcarbamoylase (ATCase). Using a diamond anvil cell at the Cornell High Energy Synchrotron Source (CHESS), we collected 2.5-Å resolution structures of ATCase at ambient temperature and pressures of 1 bar, 0.5 kbar, 1 kbar, and 1.5 kbar. Interestingly, we observe that key residues involved in substrate binding become disordered as pressure increases. We discuss what this observation reveals about allostery in ATCase, as well as the potential for HP-MX to reveal hidden conformational dynamics of enzymes.

